# The end of mass homeownership? Changes in labour markets and housing tenure opportunities across Europe

**DOI:** 10.1007/s10901-017-9551-8

**Published:** 2017-04-22

**Authors:** Rowan Arundel, John Doling

**Affiliations:** 1 Department of Geography, Planning and International Development, Amsterdam Institute for Social Science Research (AISSR), University of Amsterdam, Nieuwe Achtergracht 166, 1018 WV Amsterdam, The Netherlands; 2 Department of Social Policy and Social Work, School of Social Policy, University of Birmingham, Muirhead Tower, University of Birmingham, Edgbaston, Birmingham, B15 2TT UK

**Keywords:** Employment insecurity, Homeownership, Housing access, Labour markets, Mortgage markets, Young people

## Abstract

With continued economic growth and expanding mortgage markets, until recently the pattern across advanced economies was of growing homeownership sectors. The Great Financial Crisis (GFC) has however, undercut this growth resulting in the contraction of homeownership access in many countries and the revival of private renting. This paper argues that these tenure changes are not solely a consequence of the GFC, and therefore, reversible once long-term growth returns. Rather, they are the consequences of more fundamental changes especially in labour markets. The very financialisation that fuelled the growth of homeownership has also led to a hollowing out of well-paid, secure jobs—exactly those that fit best with the taking of housing loans. We examine longer-term declines in labour market security across Europe from before the GFC, identifying an underlying correlation between deteriorated labour market conditions and homeownership access for young adults. While variations exist across European countries, there is evidence of common trends. We argue that the GFC both accelerated pre-existing labour insecurity dynamics and brought an end to offsetting such dynamics through the expansion of credit access with the likelihood of a return to an era of widespread homeownership growth starkly decreased.

## Introduction

In most developed economies, over the last half-century or so there has been a major transformation of housing tenure patterns to what seemed, by the early years of the present millennium, to be the growing dominance of homeownership (Atterhog 2006). In each of the countries that were to become the EU25, homeownership was a minority tenure in 1945 but as a result of decade on decade increases had become, with the exception of Germany, the majority tenure by 2003. As a group—less Germany—about two-thirds of households owned their homes leading to the description of Europe as a *Union of Homeowners* (Doling and Ford 2007). The trend toward the dominance of homeownership was not confined to Europe, being characteristic across developed countries including the USA, Australia, and Canada as well as East Asia (Doling 2013). Over the last few years, however, this picture has begun to change. Homeownership rates have ceased their inexorable rise, and in some countries have gone into decline. Phrases such as *generation rent*, *boomerang kids*, and *parasite children* have gained purchase as descriptions of housing alternatives pursued by young adults who in previous decades would have been stepping onto the first rung of the homeownership ladder.

In many observations, there is an assumption, sometimes implicit, sometimes explicit, that this shift is a significant, but potentially temporary, effect of the 2007 Great Financial Crisis (GFC) and its consequences (Forrest and Hirayama 2015; Lennartz et al. 2016; McKee 2012). The implication is that once the slump, however deep and prolonged, is overcome and the business cycle picks up, things will return to usual. People will flock back to homeownership, which will again be the achievable tenure of choice for most people. The future will once more be one of mass homeownership. The argument developed in this paper is that even if the GFC may be considered as essentially no more than a downturn in the business cycle, however severe, with a (contested) expectation of a return to “normal”, the seeds of change in the dynamics of tenure choice were sown long before 2007. One particular dimension of this has been a fundamental and long-term shift in the nature of labour markets in terms of both the security of employment and financial rewards of work to growing sections of the labour market. To varying extents in different advanced economies, these changes have increasingly eroded the flow of people gaining well-paid and secure jobs, especially among younger people. Phenomena such as the *hollowing out of the middle classes*, *the polarisation of labour markets*, the rise of the *precariat*, and the crisis of *youth unemployment* all point to a reduction in the pool of people whose financial circumstances fit well with the ability to take on the long-term repayment commitment of a housing loan. While new labour markets may have little effect on the housing circumstances of long-established owners—since debt repayment has largely been realised—they have great impact on those at the beginning of their employment and housing careers. Groups who, in earlier decades, might have been expected to achieve independent living perhaps by a move into private renting as a stepping stone into homeownership, or even straight into homeownership, are now being pushed by a more precarious labour market towards alternative housing trajectories. In this world, homeownership may cease to be a housing option for the masses with countries moving closer to notions of a ‘post-homeownership’ society (Ronald 2008).

The argument presented herein challenges conceptions of recent homeownership slumps as merely a temporary and limited consequence of the financial crisis, instead uncovering the more fundamental changes in interrelated labour and property markets. The historic drivers of growing homeownership up to the GFC are initially outlined followed by a broader evaluation of the implications of the financial crisis. We then turn to empirical evidence across Europe for the sample of EU28 countries plus Norway.^1^ Firstly, the research examines descriptively longer-term declines across Europe in labour security and remuneration pointing to trends that predate the crisis years and have reduced widespread, stable middle-class jobs, especially among younger adults—fundamental preconditions to entry to homeownership—only partly masked by the expansion of credit in the pre-crisis years. Secondly, an exploratory analysis of recent ownership rates across different European countries evaluates the fundamental correlation between structural deteriorations in labour market conditions and homeownership access for young adults alongside the moderating role of the mortgage market. While countries vary in terms of total homeownership shares, labour market and mortgage conditions, and in the extent of recent declines, common trajectories are revealed. The research identifies the general—albeit not universal—tendencies toward macro-level changes in labour and housing markets. While in some countries the changes may be quite dramatic and in others more muted, together the picture reveals common processes that signify a downward impact on possibilities of homeownership growth.

## The growth of homeownership up to the GFC

An argument that the seeds of change pre-date the onset of the GFC raises the question of why—that is on what foundations—homeownership sectors commonly expanded in the preceding decades. Following the tradition established by Esping-Andersen, and Kemeny in housing studies, much of the literature on national homeownership rates has focused on cross-sectional differences. Nevertheless, a small number of studies have identified key drivers underlying the common tendency toward longitudinal increases.

Some evidence has pointed to changing demographic and household characteristics, including population ageing and tendencies toward dual incomes, being associated with higher homeownership rates (Caldera Sanchez and Andrews 2011). At the macro-level, pre-crisis economic growth and rising national prosperity, has been a determinant of the demand for homeownership. Insofar as in most developed countries homeownership is both the preferred form of tenure and contains higher quality stock than other tenures—both reflected in a strong positive correlation with income—rising demand seems likely to follow rising GDP. Rising national prosperity can be seen as increasing the pool of households with sufficient income to be evaluated by financial institutions as secure borrowers (Atterhog 2006). However, at the same time, the degree to which prosperity translates to economic capacity for a wide middle-class is certainly mediated by the distribution of wealth gains across populations (Atterhog 2006). While some expansion in prosperity likely played a role, there has not been evidence that widespread household-level labour market improvements have been the key driver of homeownership growth, especially in recent pre-crisis years.

Beyond (uneven) improvements in economic prosperity, government policies and financial transformations played an important intervening role in homeownership sector growth. Government policies have directly influenced the possibilities and attractiveness of different tenure choices (see Atterhog 2006; Scanlon and Whitehead 2004). In many countries these include policies enhancing the attractiveness of owning through, for example, direct grants for house-buying, mortgage guarantee schemes, and tax relief on interest payments, capital gains and imputed rent. Legislation allowing the privatization and sale of social housing to increase homeowners has long been in place in some countries, such as Australia and the UK (Forrest and Hirayama 2009, 2015; Beer et al. 2007) and, since the 1980s, expanded to most of the countries with a significant stock (Scanlon 2014).^2^ In contrast, housing allowances for renters, rent-control and tenure security regulations have increasingly been eroded under the impetus of a neo-liberal political project that has emphasized homeownership as a widespread housing solution (Forrest and Hirayama 2009, 2015; Andrews and Caldera Sanchez 2011).

**Fig. 1:**
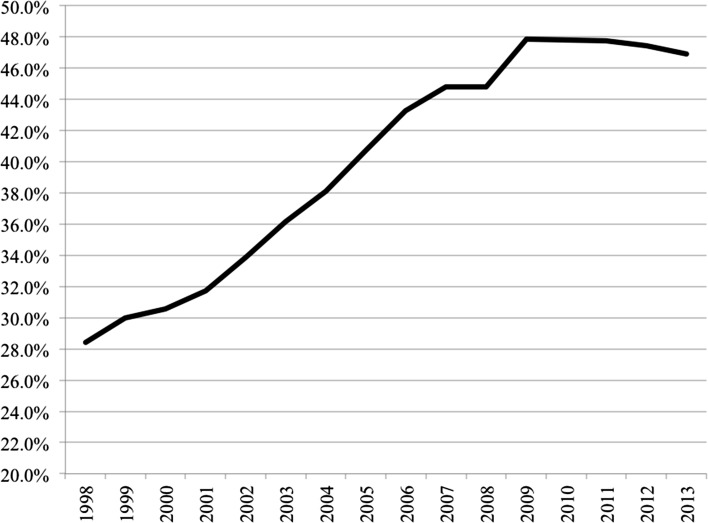
Residential mortgage-dept-to-GDP ratio across Europe. Includes all EU15 countries Austria, Belgium, Denmark, Finland, France, Germany, Greece, Ireland, Italy, Luxembourg, Netherlands, Portugal, Spain, Sweden, UK along with Bulgaria, Cyprus, Estonia, Latvia, Lithuania, Malta, Poland, Slovenia plus Norway. (Data for Croatia, Czech, Hungary, Romania, Slovakia, unavailable for this measure). *Note*: measures are population weighted. *Data Source*: European Mortgage Federation, Hypostat 2014 Database

Fundamental drivers of homeownership in the pre-crisis period are tied up with processes of *financialisation*. While a broad and sometimes vague concept—encompassing the increasing dominance of financial markets and institutions in the operation of the economy (Aalbers 2008)—financialisation describes real forces that have progressively altered the nature of housing, through commodification, neoliberal re/deregulation, and the integration of property with flows of capital and debt (Fernandez and Aalbers 2016; Aalbers 2008). In these ways, property markets have been characterised by intensified flows of investment—embedded in global circuits of capital—alongside the dramatic rise of mortgage credit. As part of these wider trends, over the last 20 years, and in some countries even longer, mortgage markets have been progressively liberalised. Central banks have reduced controls over lending, sometimes bringing to an end independent housing finance circuits, and allowing a wider range of institutions to be involved. Competition has been increased reducing costs to consumers; with product innovation making mortgages more accessible to a wider group of potential borrowers (Andrews and Caldera Sanchez 2011; Aalbers 2012). Crouch has argued that such an expansion of credit-driven growth represented a major shift towards a policy regime of *privatised Keynesianism* where “instead of governments taking on debt to stimulate the economy, individuals did so” (Crouch 2009: 390). An outcome of these trends is measurable by the growth in Europe of residential mortgage debt across developed economies throughout the 1990s and up to the mid 2000s (see Fig. 1) reflecting the increasing ability of households to access housing loans (Scanlon and Whitehead 2004). Looking across European countries (shown in Table 1), residential debt levels expanded in the pre-crisis period across every country, except Germany, albeit varying in absolute levels and rates of growth. Such credit growth increased general domestic demand in many countries (Crouch 2009) and, in housing markets, resulted in a tendency for households to be less frequently forced, in advance of becoming homeowners, to save from their income or to borrow money from their families and friends. Being increasingly able to obtain loan finance, homeownership became more accessible to households with incomes further down the income distribution, which also meant further down the age distribution.

**Table 1:** Descriptive housing and labour market statistics across Europe by country

	Housing tenure shares among 18–34 year olds				Mortgage market measures			Labour market measures				
	Homeownership		Rental	Coresidence with parent (s)	Average residential mortgage-debt-to-GDP			Gini coefficient of equivalised disposable income		Adjusted wage share as % of GDP		
	2013^c^	Post-crisis annual rate of change 2007–2013	Post-crisis annual rate of change 2007–2013	Post-crisis annual rate of change 2007–2013	2012^c^	Pre-crisis annual rate of change 1998–2007	Post-crisis annual rate of change 2007–2014	2012^c^	Annual rate of change 2000–2014	2012^c^	Annual rate of change 1960–2017 (EU15)	Annual rate of change 1995–2017
Units^b^	%	pp	pp	pp	%	pp	pp	%	pp	%	pp	pp
Austria	16.28	−0.65	0.46	0.19	28.1	1.13	0.68	30.5	0.26	62.81	−0.22	−0.19
Belgium	31.36	−0.09	−0.45	0.54	48.9	1.14	2.12	27.6	−0.29	67.79	0.08	−0.10
Bulgaria	27.50	−0.56	−0.16	0.72	8.9	1.06	−0.18	36.0	0.74	57.39	–	0.40
Croatia	21.50	–	–	–	19.1	–	–	32.0	–	71.63	–	−0.11
Cyprus	28.90	−0.46	0.68	−0.22	71.6	4.57	4.52	34.3	0.42	60.55	–	−0.04
Czech	31.78	−0.08	−0.27	0.34	14.2	–	–	26.1	0.01	54.01	–	0.11
Denmark	25.93	−2.17	2.15	0.01	94.5	1.98	0.17	29.1	0.44	64.90	−0.10	0.11
Estonia	39.97	−0.38	0.62	−0.24	39.5	3.62	0.13	33.2	−0.03	55.96	–	−0.05
Finland	35.53	−0.23	0.32	−0.09	44.9	1.80	0.00	27.1	0.11	65.18	−0.24	0.05
France	26.03	0.18	−0.97	0.79	42.8	1.66	1.48	33.1	0.09	66.93	−0.17	0.03
Germany	13.89	−0.15	0.35	−0.21	44.4	−0.47	−0.58	30.1	0.41	62.84	−0.19	−0.06
Greece	16.44	−0.75	0.32	0.42	38.6	2.66	1.47	36.7	0.11	58.89	−0.12	0.09
Hungary	26.18	−2.22	0.09	2.12	20.6	–	–	30.6	0.19	56.70	–	−0.45
Ireland	20.37	−0.58	1.73	−1.15	59.5	5.42	−2.92	32.5	0.06	50.90	−0.45	−0.64
Italy	19.51	−0.88	0.15	0.73	23.3	1.33	0.57	35.2	0.24	61.96	−0.15	0.07
Latvia	34.67	−0.09	0.65	−0.56	24.1	3.67	−2.00	35.5	0.11	49.45	–	0.05
Lithuania	40.24	0.76	−0.20	−0.56	17.6	1.84	−0.20	35.2	0.29	48.61	–	0.06
Luxembourg	30.44	0.07	−0.71	0.63	50.6	1.69	2.15	34.8	0.19	59.30	0.20	−0.07
Malta	33.05	–	–	–	45.0	3.29	1.32	–	–	57.68	–	−0.22
Netherlands	33.48	−1.11	0.72	0.40	108.9	4.20	1.05	28.0	−0.20	65.95	−0.19	−0.13
Norway	51.70	0.33	−0.76	0.42	67.6	1.52	1.80	25.9	−0.22	51.77	–	−0.32
Poland	30.89	0.79	0.22	−1.01	20.8	1.13	1.50	32.4	0.06	54.07	–	−0.61
Portugal	24.48	−0.83	0.55	0.28	66.9	2.80	0.37	36.0	−0.11	61.45	−0.31	−0.36
Romania	36.06	−0.90	0.07	0.83	6.7	–	–	27.3	0.41	54.43	–	−0.66
Slovakia	21.85	−0.34	0.11	0.22	19.3	–	–	26.1	–	48.08	–	0.10
Slovenia	22.25	0.15	0.68	−0.83	14.9	0.86	1.17	25.6	0.21	72.53	–	−0.37
Spain	27.32	−1.21	1.33	−0.12	62.3	3.86	−0.25	35.9	0.19	60.94	−0.17	−0.22
Sweden	35.90	−1.05	0.30	0.75	82.1	1.39	3.98	27.3	0.17	63.21	−0.16	0.20
UK	21.81	−1.99	2.56	−0.57	80.8	3.97	−0.95	32.6	−0.03	66.34	−0.06	0.11
European average^a^	24.73	−0.63	0.53	0.10	47.4	1.82	−0.35	30.6	0.19	61.71	−0.14^d^	−0.04
Source	EU−SILC, authors’ calculations				European Mortgage Federation			Eurostat		AMECO		

	Labour market measures								
	Share of involuntary part-time workers in total employment			Employment participation among 15–24 year olds		18–25 year olds with income under 50% of median^f^		Labour force share of 15–24 years olds ‘not in permanent employment’^e^	
	2012^c^	Annual rate of change 2000–2014 (EU15)	Annual rate of change 2005–2014	2012^c^	Annual rate of change 1992–2014	2012^c^	Annual rate of change 2004–2012	2012^c^	Annual rate of change 2000–2014
Units^b^	%	pp	pp	%	pp	%	pp	%	pp
Austria	2.51	0.09	0.07	53.67	−0.32	0.10	0.25	41.79	0.39
Belgium	2.34	−0.16	−0.13	25.26	−0.37	0.11	0.13	44.81	0.45
Bulgaria	–	–	–	–	–	–	–	34.90	−0.96
Croatia	–	–	–	–	–	–	–	43.49	0.27
Cyprus	–	–	–	–	–	–	–	–	–
Czech	1.20	–	0.03	25.17	−0.90	0.05	0.09	41.36	0.70
Denmark	4.35	0.09	0.06	55.00	−0.43	0.22	0.39	29.41	−0.30
Estonia	–	–	–	33.70	−0.58	0.14	0.18	52.57	−0.18
Finland	3.18	0.01	0.06	43.33	0.16	0.15	−0.19	57.39	0.05
France	5.27	0.18	0.24	28.38	−0.13	0.14	0.28	66.68	1.17
Germany	3.91	0.10	−0.13	46.62	−0.41	0.12	−0.14	32.03	−0.06
Greece	4.83	0.31	0.44	13.02	−0.69	0.22	1.31	44.48	1.11
Hungary	2.72	–	0.18	18.45	−0.54	0.11	0.40	56.55	2.08
Ireland	9.04	0.44	0.81	27.90	−0.39	0.11	0.29	69.72	1.89
Italy	9.71	0.61	0.75	20.39	−0.48	0.15	0.40	33.74	−0.81
Latvia	–	–	–	–	–	–	–	55.84	1.56
Lithuania	–	–	–	–	–	–	–	45.84	2.00
Luxembourg	2.39	0.10	0.05	21.75	−1.32	0.08	−0.36	29.01	1.05
Malta	–	–	–	–	–	–	–	30.56	−0.47
Netherlands	3.91	0.24	0.32	63.32	0.08	0.20	0.29	75.15	1.16
Norway	1.57	–	−0.12	52.65	0.09	0.30	0.78	73.20	2.10
Poland	1.95	–	−0.09	24.68	−0.30	0.11	−0.71	26.63	0.11
Portugal	5.11	0.17	0.25	23.02	−1.03	0.16	0.79	–	–
Romania	–	–	–	–	–	–	–	46.62	0.42
Slovakia	2.64	–	0.26	20.11	−0.57	0.07	−0.19	56.62	0.47
Slovenia	–	–	–	–	–	0.07	0.21	66.19	0.75
Spain	9.12	0.61	0.71	20.25	−0.74	0.17	0.79	66.32	0.19
Sweden	8.11	0.28	0.46	40.04	−0.43	0.18	0.39	82.15	0.66
UK	4.90	0.15	0.28	49.58	−0.47	0.20	0.54	33.04	0.35
European average^a^	4.95	0.27^d^	0.22	34.47	−0.42	0.15	0.24	46.04	0.32
Source	OECD			OECD		OECD		OECD	

^a^ Population weighted, excluding missing data countries. For occasional instances of missing years in the data, values interpolated based on trendline (only where enough years of data allow interpolation)

^b^ *pp* refers to percentage points of change

^c^ Absolute values across countries are displayed for 2012 as this year is available across all measures and reflects variables used for subsequent regression models. The only exception is homeownership rates for 2013 as these values are also used in the regression modeling and better represent housing outcomes in the subsequent year following 2012 measures

^d^ Average for EU15 countries only

^e^ Not in permanent employment includes those unemployed, in ‘casual’ employment, or with short-term contracts of less than a year in duration

^f^ After all taxes and transfers

## Subprime lending and the financial crisis

While the alignment of some or all of these factors pointed to sustained increases in the size of homeownership sectors across many advanced economies, the financial crisis of 2007 marked a stark coming to terms with the sustainability of such growth. The GFC was undoubtedly a crisis of housing both in its origins and its outcomes, with further repercussions across the global economy. From the outset it was apparent that the origins of the crisis were to be found in one of the very factors that had stimulated, or sustained, homeownership growth. In the 2000s, financial institutions, especially but not exclusively in the USA, had promoted sub-prime mortgages—that is loans to households whose income position was not sufficiently robust to ensure a high probability of meeting repayment schedules. In turn, this was supported by mortgage-backed securities that aggregated loans and sold them onto banks in many countries, thereby both hiding and spreading risks (Aalbers 2012; Aalbers and Christophers 2014). As the extent of toxic loans—both in total size and permeation throughout financial institutions—became apparent and the viability of many financial institutions came into question, the amount of lending to those seeking to buy homes decreased. This is evidenced by the average trends in mortgage debt (Fig. 1) where the expansion of loans from the late 1990s is clearly reversed following the 2007 crisis. With only a few exceptions, a general pattern of either slowed growth or shrinking debt levels is reflected across the sample of European countries in the post-crisis period (Table 1). As the crisis in banking began to bite, the impact quickly spread to real economies and throughout the developed world, demand and production decreased, unemployment rose and GDP slowed or in some countries fell. Not only did banks often have less money to lend in the housing sectors than before 2007, but fewer households were in a position to seek housing loans anyway.

**Fig. 2:**
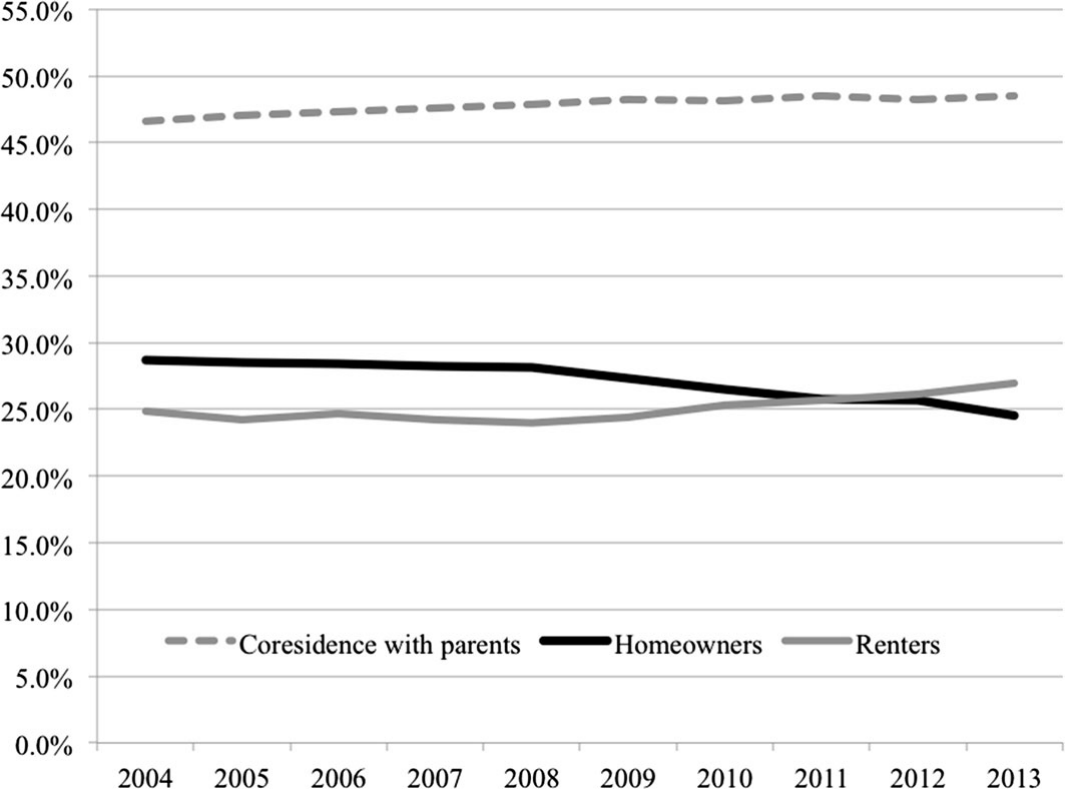
Housing tenure positions among 18–34 years olds across Europe. Includes all EU15 countries Austria, Belgium, Denmark, Finland, France, Germany, Greece, Ireland, Italy, Luxembourg, Netherlands, Portugal, Spain, Sweden, UK along with Bulgaria, Cyprus, Czech Republic, Estonia, Hungary, Latvia, Lithuania, Poland, Romania, Slovakia, Slovenia plus Norway. (Data for Croatia, Malta unavailable for this measure). *Note*: measures are population weighted. *Data Source*: Eurostat EU-SILC 2004–2013, authors’ own calculations

In this harsher economic context, former drivers of the expansion of homeownership were undercut. Across most European countries there have been marked reversals of earlier growth. Looking at country-level trends post-crisis (Table 1)—barring a few exceptions that saw some continued growth (Poland, Lithuania and to a lesser extent Norway) or limited cases of stagnation—the data reveal a majority of the countries experiencing decreasing access to homeownership among young adults. In some countries, this has resulted in a rise in private rental, while others have seen more prominent shifts towards growing shares of young adults in parental co-residence (for more detailed analyses of post-crisis tenure trajectories across European countries see Lennartz et al. 2016). Even pre-crisis, close examination of tenure shares for a range of developed countries indicated that in a majority the trends were stabilising, particularly in terms of slowed entry of young adults into homeownership (Scanlon and Whitehead 2004). These trends prompted Scanlon and Whitehead (2004) to pose the question of whether the tenure “had reached a plateau”, albeit a plateau at different heights in different countries. Examining general tenure dynamics across the sample of European countries over recent years (Fig. 2), diminished entry into homeownership is clearly apparent with larger proportions of young adults either staying in—or returning to—the parental home or moving into the rental sector. The average data trend reveals gradual homeownership declines even in the pre-crisis years that sharply accelerated post-GFC. As discussed previously, much of the flow that did occur into homeownership was predicated on expanded credit, supportive government policies, and relaxed mortgage restrictions. Given the dramatic growth in credit, it is therefore all the more striking that declines or stagnation are already apparent in the pre-GFC ‘boom’ years.

Notwithstanding the stark outcomes of the GFC, there is often a lack of understanding of the true significance of the financial crisis within the context of longer-term economic and housing trends. Taking on a broader perspective, the GFC was not the clear ‘reversal of homeownership expansion’ as it has been characterised, but rather the salient failure of ‘market solutions’ to broader structural problems within highly-financialised homeownership and labour markets. Crouch (2009) argues that, while in the pre-crisis period expanding private (housing) debt supported by unregulated derivatives markets provided an attempt to reconcile problems of neoliberalisation in the labour market—under what he termed ‘privatised Keynesianism’—the GFC clearly demonstrated the unsustainability of such a policy regime. In other words, the subprime mortgage crisis quashed hopes that growing labour market insecurity impacts on homeownership opportunities could be resolved through ever-expanding credit access and complex financial products.

While the tremendous economic repercussions of the GFC are evident, especially in housing outcomes, the broader lesson lies in the inherent contradiction of an increasingly marketized homeownership provision system wherein interrelated processes of financialisation and neo-liberal re/deregulation undermine the very pre-conditions needed for property purchase. As developed countries come through the present downturn, their economies quicken, employment and confidence returns, and financial institutions become more robust, will normal business be resumed? Will homeownership sectors once again everywhere increase in size? Such questions raise the issue of what is meant by ‘normal’ and especially whether mortgage markets—and more generally financial markets—will return to anything like their former characteristics. The structural causes of the crisis, however, undercut a return to mortgage credit expansion for growing sectors of economically precarious populations. Unsurprisingly, the crisis has instead seen the development of new architectures of financial regulation. Writing in 2010 one observer remarked that “the world is now knee-deep in proposals to reform finance” (The Economist 2010), with national governments, the EU and international organisations all active. As one example, the Financial Stability Board (FSB)^3^ has recommended minimum underwriting standards intended to limit the risks posed by mortgage markets to wider financial stability. While the new architecture is not fully in place in all developed countries, it is likely that the future will be characterised by financial institutions with greater capital and liquidity requirements with the emphasis in many countries being switched from growth in new lending toward stability and safety.

## Longer-term labour market trends

Notwithstanding the alignment of certain factors promoting increases in homeownership sectors, even in the early years of the present millennium there were signs that such a future was not assured. Longer-term economic restructuring destabilized elements of economic certainty that existed under previous Fordist conditions (see Beck 1992; Giddens 1999). On the one hand, technological change facilitated the replacement of workers with machinery—increasingly beyond the traditional manufacturing sectors (Brown et al. 2011)—and many of them in the middle income distribution (OECD 2011a, b). At the same time, globalisation and the increasing mobility of capital and labour has meant that companies have had possibilities for reducing the cost of their labour input through relocating to low-wage economies, thus, also reducing unionized bargaining power. Dimensions of globalisation and financialisation have broadly proceeded in tandem where neoliberal policies have seen global integration impacting both the nature of labour and financial networks. Indeed, assessing econometric developments across 28 advanced economies, Stockhammer (2013) asserts that processes related to globalisation and financialisation have been the most important causes of the changing share of economic gains accruing to labour. Such processes have included the opening up of more investment options to firms that have been able to invest in financial assets and to choose to relocate abroad, the empowering of shareholders that has aligned management interests towards short-term profits, alongside the availability of credit that has allowed lower-income workers to continue to consume. Such labour market and mortgage finance transformations have been driven by policy choices which have seen a certain level of congruence in political economy approaches across countries—what Aalbers (2015) argues constitutes part of a wider shift towards a ‘late neoliberal’ regime. Taken together, the measurable outcomes of such changes have been felt in the labour market in terms of higher unemployment, underemployment, contract insecurity or increasing disparities in labour outcomes (Buchman and Kriesi 2011; McKee 2012; Nolan et al. 2014). The broad effect has been to reduce the proportion of national populations that have well-paid and secure jobs, particularly among young people at the start of their work and housing careers.

This paper brings into focus evidence of crucial longer-term changes in European labour markets and their effect in shaping tenure trends; looking at rates of income inequality, the changing share of wages accruing to labour, employment insecurity measures as well as poverty and employment participation rates among younger cohorts. Using macro-level data from across the EU28 plus Norway, the evidence points to longer-term shifts towards decreasing proportions of those in secure and well-paid employment. These measures are presented descriptively in Table 1 across countries with average longer-term dynamics for Europe presented in subsequent figures.^4^ While important country-level variations are acknowledged, the research focuses on common macro-level trends in changing labour and housing markets. The evidence points to labour market conditions which have changed markedly since, in fact, some years before the onset of the GFC and provide an explanation for already apparent slowdowns in many homeownership sectors, further exacerbated by post-crisis developments.

### The erosion of the middle-classes

**Fig. 3:**
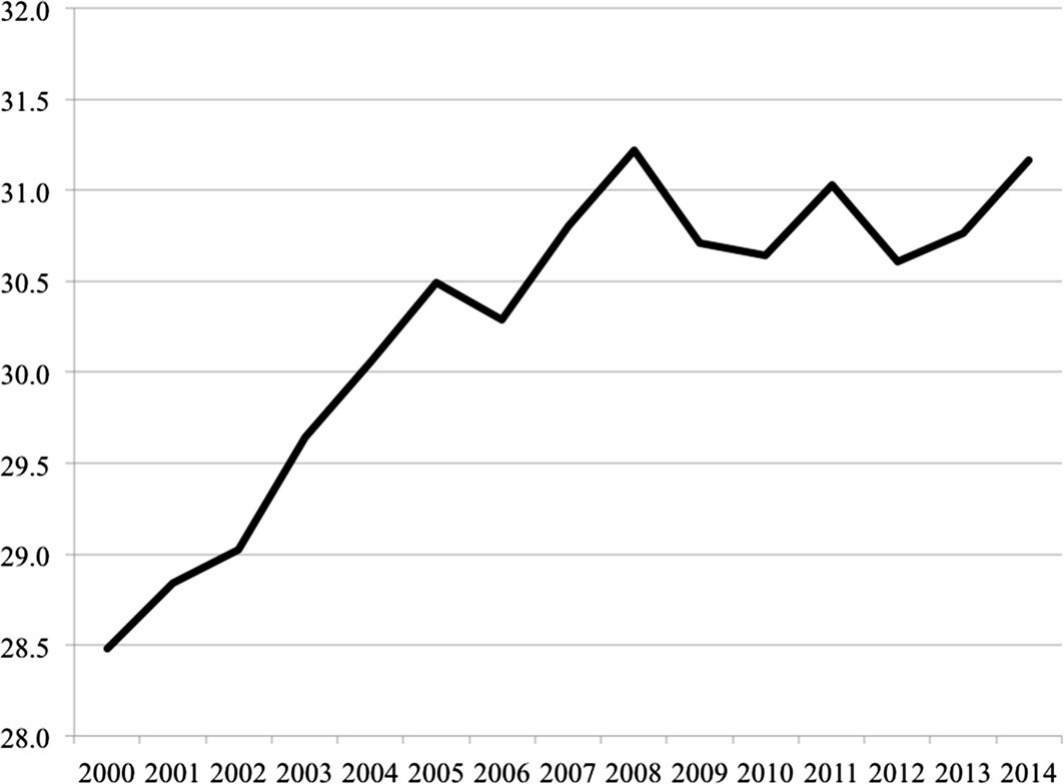
Gini coefficient of equivalised disposable income across Europe. Includes all EU15 countries Austria, Belgium, Denmark, Finland, France, Germany, Greece, Ireland, Italy, Luxembourg, Netherlands, Portugal, Spain, Sweden, UK along with Bulgaria, Cyprus, Czech Republic, Estonia, Hungary, Latvia, Lithuania, Malta, Poland, Romania, Slovenia plus Norway. (Data for Croatia, Slovakia unavailable for this measure). *Note*: measures are population weighted. *Data Source*: OECD

In recent decades, labour markets across developed countries have been characterised by processes of polarisation: the proportion of jobs in middle income ranges has declined with a diversion downwards to low-paid jobs and upwards to high-paid jobs. Goos et al. (2009) use data from the European Union Labour Force Survey to identify employment change by occupational type in the older member states (EU15) plus Norway over the period 1993–2006. Corroborating accounts from the USA, the data points to distinct polarisation across these 16 countries with the highest and lowest paid occupational groups increasing their share of employment by 6 and 2% points respectively, while the medium-paid group decreased by 8% points (Goos et al. 2009). Direct measures of inequality across Europe reinforce the picture of increasing disparity. Looking at country-level data (Table 1), the pattern reveals a clear increase in income inequality across a majority of European countries—with only a few exceptions (Belgium, Netherlands, Norway and Portugal) or a few seeing no clear change. Examining average national Gini coefficients measuring the distribution of equivalised disposable income across the EU28 plus Norway (Fig. 3), there is a clear pattern towards income polarization across these economies. Importantly, this trend was most prominent before the GFC with income inequality appearing to plateau in the post-crisis period albeit at a historically high level.

This trend towards polarisation has prompted narratives about the *squeezed middle* and a *hollowing out of the middle classes,* indicating the decline in middle-range workers who formerly could rely on steady incomes to support climbing the housing ladder. The polarisation results in smaller proportions of labour forces in this position, while increasing both the shares with enhanced options to consume and invest and those having reduced options for either. As Atterhog has pointed out, GDP is not in itself sufficient as an indicator of demand, since this requires also taking “the distribution of … income into consideration” (2006, p.11). His argument is that high income inequality is likely to lead to small numbers of households able to afford property, while, conversely, more equal distributions favour more homeownership.

### Wage share

**Fig. 4:**
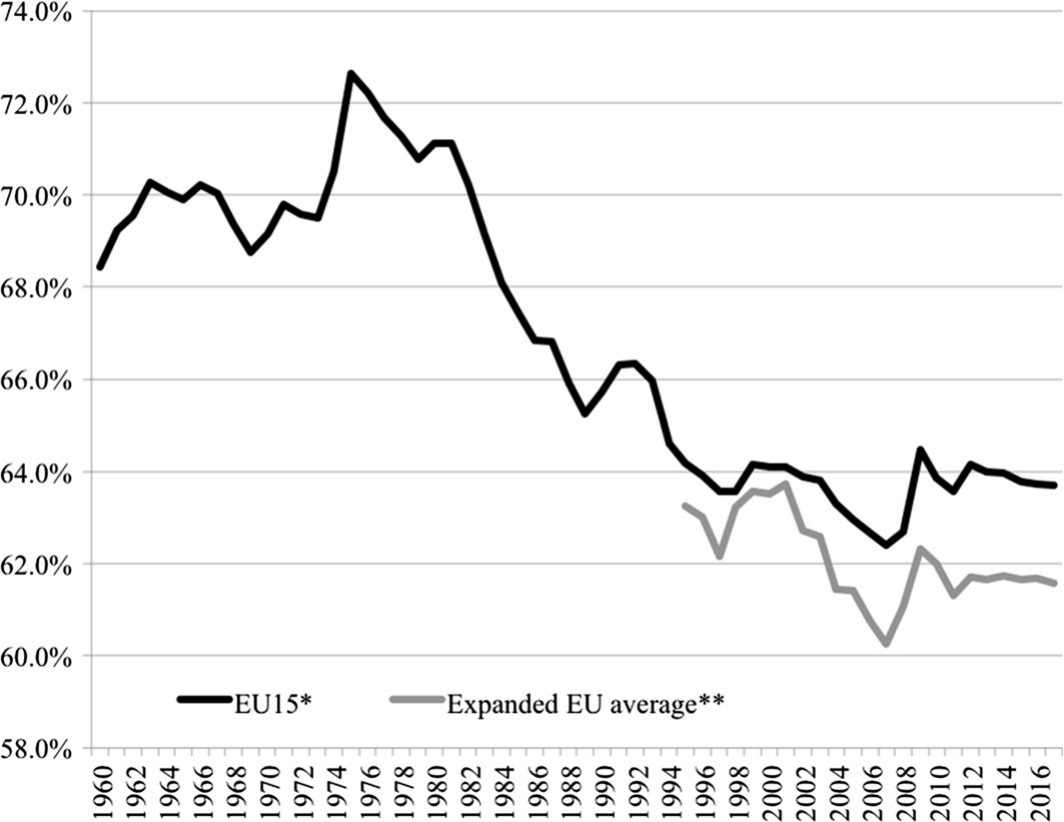
Adjusted wage share as % of GDP. * EU15 countries include: Austria, Belgium, Denmark, Finland, France, Germany, Greece, Ireland, Italy, Luxembourg, Netherlands, Portugal, Spain, Sweden, UK. ** includes EU15 along with Croatia, Bulgaria, Cyprus, Czech Republic, Estonia, Hungary, Latvia, Lithuania, Malta, Poland, Romania, Slovakia, Slovenia plus Norway. *Note*: measures are population weighted. *Data Source*: European Commission, AMECO Database

Polarisation has been accompanied by a broader underlying macro-level trend further exacerbating economic opportunity. It was once a truism held by economists that under productivity gains labour achieved a roughly constant share of national income. The wage share is an approximate measure of the distribution of income between capital and labour—the latter consisting of both personal income and welfare benefits. Figure 4 indicates developments across the core EU15 economies as well as for the expanded European sample where data is available. From the post-war decades until the beginning of the 1980s, the wage share generally remained stable in advanced economies so that the benefits of economic growth were consistently split between capital and labour. Since the 1980s, however, that historical relationship has been fractured with labour taking smaller and smaller shares. Again, baring a couple of exceptions, these patterns of declining wage shares are replicated across European countries (Table 1), with only Croatia and Slovenia remaining in 2012 over the 70% mark, or near the historical equilibrium expected in traditional economic theory. In essence, the productivity gains that have generated increases in GDP per capita have not been translated into the same rises in pay and other compensations for labour as in previous decades, with greater shares accruing to owners of capital. Looking at the European average, the data reveals a stark reduction in labour shares from over 72% down to the low 60s in recent years and is another crucial indicator of the long-term erosion of a strong middle-class and on-going concentration of economic gains among (certain) holders of capital.

### Employment insecurity

**Fig. 5:**
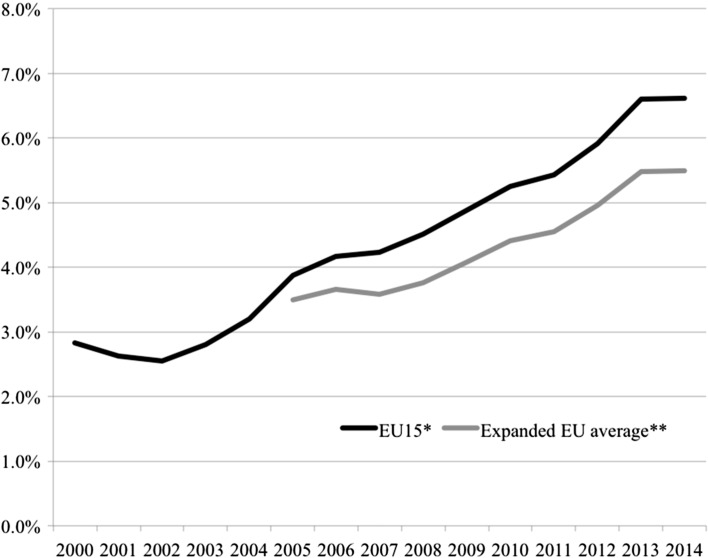
Share of involuntary part-timers total employment. * EU15 countries include: Austria, Belgium, Denmark, Finland, France, Germany, Greece, Ireland, Italy, Luxembourg, Netherlands, Portugal, Spain, Sweden, UK. ** includes EU15 along with Czech Republic, Hungary, Slovakia, plus Norway. (Data for Croatia, Bulgaria, Cyprus, Estonia, Latvia, Lithuania, Malta, Poland, Romania, Slovenia unavailable for this measure). *Note*: measures are population weighted. *Data Source*: OECD

Beyond significant proportions facing reduced economic capacity with rising income disparity and decreasing labour shares of capital, the nature of employment has also changed resulting in fewer households having the type of stable and full-time work usually associated with homeownership. As an indicator of precarious employment, the share of involuntary part-time workers in the workforce (Fig. 5) indicates increasing proportions across Europe since the early 2000s. Again, the average trend reflects increases even during the pre-GFC ‘boom’ years. Despite some variation at the country-level with certain cases only experiencing moderate upturns and a few limited decreases, the majority have seen substantial increases (see Table 1)—most prominently in southern European countries as well as Sweden, Ireland, the UK, France and the Netherlands. More precarious labour conditions often exclude possibilities for accessing mortgage financing as well as impacting the decision to commit to home purchase when the need of moving for employment may be anticipated (Coulson and Fisher 2002). While unemployment levels have tended to fluctuate greatly with economic cycles, evidence shows that, even when employment opportunities return, positions are less likely to be full-time or stable.

**Fig. 6:**
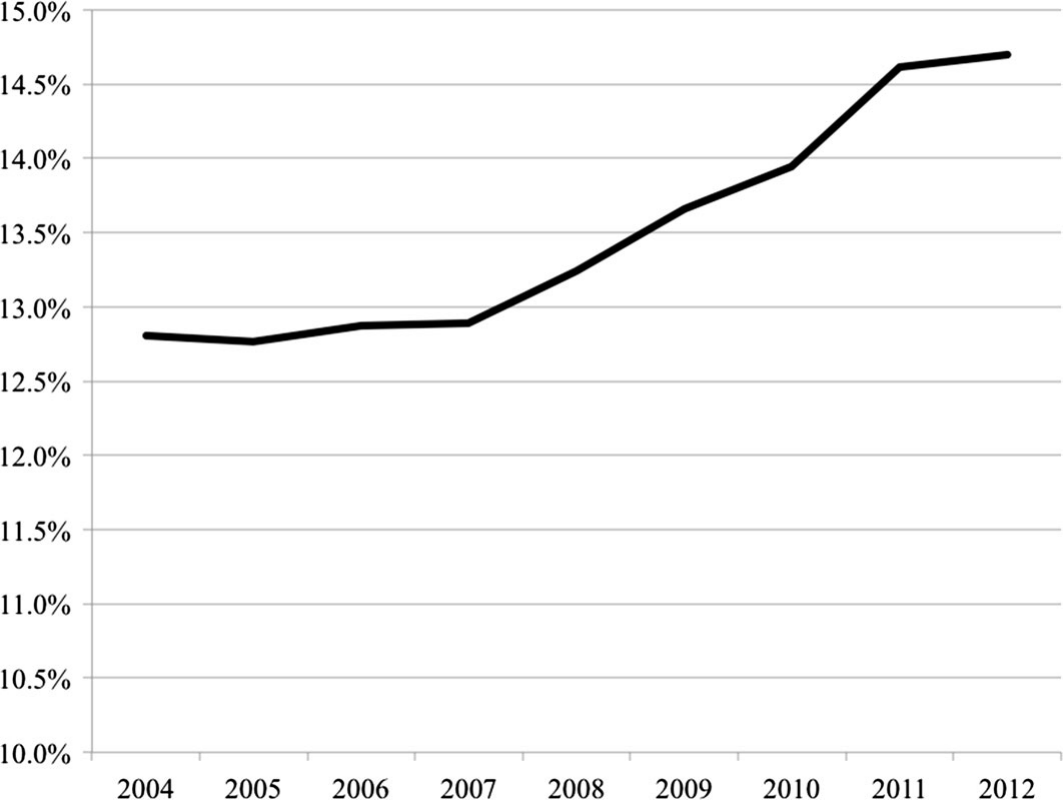
Proportion of 18–25 year olds with incomes below 50% median across Europe. Includes all EU15 countries Austria, Belgium, Denmark, Finland, France, Germany, Greece, Ireland, Italy, Luxembourg, Netherlands, Portugal, Spain, Sweden, UK along with Czech Republic, Estonia, Hungary, Poland, Slovakia plus Iceland, Norway. (Data for Croatia, Bulgaria, Cyprus, Latvia, Lithuania, Malta, Romania, Slovenia unavailable for these measures). *Note*: measures are population weighted. *Data Source*: OECD

Outcomes of economic restructuring and interrelated labour market trends have not affected all groups equally and, within inequality dynamics, a significant intergenerational dimension has been apparent. While older cohorts have tended to benefit from relatively favourable past economic conditions, are more shielded from economic restructuring and often have been able to accumulate (property) wealth, labour market changes have had the clearest adverse impacts on young adults. Existing evidence from the US and the UK show a decrease in relative incomes of younger people since before the crisis (Andrew and Pannell 2006; Bell and Blanchflower 2011), while recent labour market difficulties have further most affected younger generations (Hills et al. 2013; Clapham et al. 2010; McKee 2012). Looking at the European data, there has been a meaningful increase in the proportion of 18–25 year olds^5^ with incomes below 50% of the national median since 2000 (see Fig. 6). Other than a few exceptions, country-level data (Table 1) reveals common trends of increasing shares of young adults with low-incomes across most contexts. These patterns show a significant and growing proportion at the age of emerging adulthood independence with clearly limited opportunities in saving up for current or future homeownership.

**Fig. 7:**
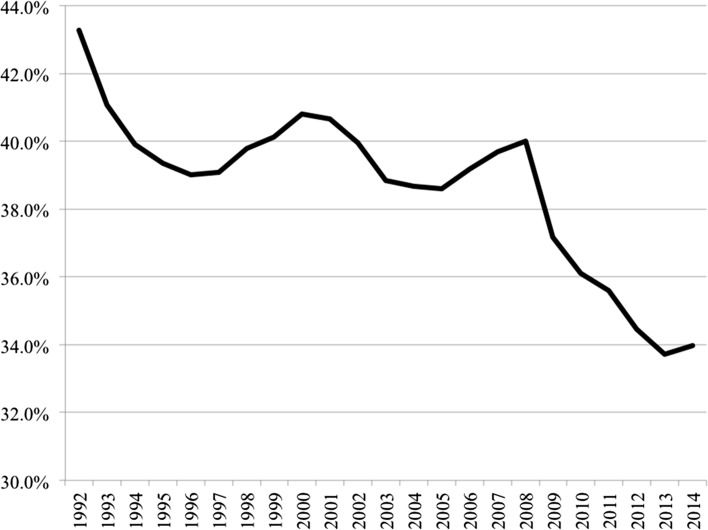
Employment participation among 15–24 year olds across Europe. Includes all EU15 countries Austria, Belgium, Denmark, Finland, France, Germany, Greece, Ireland, Italy, Luxembourg, Netherlands, Portugal, Spain, Sweden, UK along with Czech Republic, Estonia, Hungary, Poland, Slovakia plus Iceland, Norway. (Data for Croatia, Bulgaria, Cyprus, Latvia, Lithuania, Malta, Romania, Slovenia unavailable for these measures). *Note*: measures are population weighted. *Data Source*: OECD

Worsening economic trends among young adults are further compounded by other labour and policy dimensions. For example, growing demands for a better-educated workforce combined with government spending cuts and rising education costs in many countries have meant delayed entry into the labour market and often substantial debts upon graduation, especially among those with more disadvantaged backgrounds (Buchmann and Kriesi 2011; Hills et al. 2013). Eventual entry into labour markets has subsequently often been met with diminished opportunities. Across the OECD countries, 5.1% of workers aged 15–24 were unemployed in 1970, while this stood at 20.9% in 2009 (Bell and Blanchflower 2011) and, in 2015, remained at 20.4% among the EU28 countries (OECD 2016a, b). While unemployment rates have tended to fluctuate greatly following economic cycles (OECD 2016a, b), the total employment rate reflects the long-term trend of diminishing proportions of young adults in the workforce. The employment rate, beyond unemployment measures, captures increases in education enrolment, choices to extend education, as well those who have for various reasons given up on searching for work—all often linked to the lack of foreseeable labour market opportunities (Buchmann and Kriesi 2011; Clark 2011). The average trend (see Fig. 7) shows long-term decreases in employment participation among 15–24 year olds across European economies stretching back to at least 1990 and bucking trends among older age groups which saw increases in participation (OECD 2016b). Again, looking across countries (Table 1), most reflect substantial decreases in employment participation among young adults with only a few exceptions. While extended education can also be interpreted as primarily a delay of labour market entry, there is evidence that such postponements have significant correlations with diminished future wages and unemployment (Bell and Blanchflower 2011; Clark 2011).

Taken together, trends in delayed labour market entry, increased educational indebtedness, and a lack of well-paid and stable job opportunities for recent generations have hindered traditional adulthood transitions including in housing trajectories (see Arundel and Ronald 2016) with diminished abilities in meeting loan requirements and accessing homeownership. These inter-generational divisions compound broader changes in employment insecurity, the polarisation of income, and the declining share of productivity gains accruing to labour.

## Employment insecurity, mortgage and homeownership access

In order to aid understanding of the relationship between employment insecurity and homeownership opportunities, the second empirical investigation examines the interaction between labour market conditions and homeownership rates. Looking at 28 countries across the expanded European Union (plus Norway) provides a useful sample with variation in labour market conditions for younger adults, young homeownership entry rates and levels of mortgage credit access. While the analysis remains exploratory and the data does not allow a long-term longitudinal analysis, variation that does remains across countries (see descriptive statistics in Table 1) captures cross-sectional differences and may further inform differences that occur over time. Fundamentally, the analysis across European countries helps to understand the underlying relations between contemporary levels of employment security and homeownership outcomes alongside the role of mortgage credit markets. The outcome of recent homeownership attainment among young adults is examined through a measure of homeownership shares among 18–34 years old in 2013. Tested measures of labour market insecurity include: recent changes in the proportion of 18–25 year olds below 50% of median income, the Gini coefficient of equivalised income, and the labour force share of 15–24 year olds ‘not in permanent employment.’^6^ As a proxy for the marketization of the housing system and access to mortgage credit, a measure of mortgage debt to GDP is examined. Finally, the base ‘natural’ homeownership level is controlled for through average national homeownership shares among all adults over the 2006–2012 period. Due to the limited sample size, regression models are subsequently performed separately for each of the labour market variables (Table 2—models a, b and c).

**Table 2:** Regression models with outcome: homeownership rate among 18–34 year olds 2013

	(a)	(b)	(c)
Change in low-income rate among 15–24 year olds 2007–2012	−1.552 (2.98)**		
Gini coefficient of equivalised disposable income 2012		−0.57 (−1.8)	
Labour force share of 15–24 year olds ‘not in permanent employment’ 2012			−0.164 (2.46)*
Mortgage debt to GDP ratio 2012	0.166 (3.56)**	0.126 (2.71)*	0.13 (2.83)**
Average homeownership rate 2006–2012	0.973 (5.67)**	0.798 (4.96)**	0.715 (4.50)**
Constant	−41.975 (3.52)**	−14.142 (−0.91)	−17.864 (−1.37)
*R* ^*2*^	0.67	0.54	0.58
N	22	28	28

Quantities in (.) are t statistics. * *p* < 0.05, ** *p* < 0.01

The results (see Table 2) provide evidence supporting the expected relationship between labour market insecurity measures and reduced homeownership access among young adults—taking into account mortgage marketization levels and base homeownership levels. Model (a) reveals that increasing shares of young adults (18–25) with income levels below 50% of the median are strongly correlated with relatively lower levels of young homeownership (*p* < 0.01). Secondly, model (b) provides some support—albeit only moderately significant (*p* = 0.08)—for the association between higher total income inequality levels and lower homeownership rates seeming to corroborate notions of a hollowing out of the middle class undermining widespread ownership potential (i.e. Atterhog 2006). Finally, the labour force share of young adults not in permanent employment is significantly associated (*p* < 0.05) with lower homeownership levels (model c). This supports the importance of employment term stability, which would affect accessing mortgage loans beyond absolute income as well as motivations for housing commitments when labour instability might necessitate future moves.

While remaining an indirect proxy, the mortgage-debt-to-GDP ratio reflects the expectation that countries with more developed and accessible mortgage markets display more opportunity for access to homeownership. While countries that retain more marketised mortgage markets are associated with overall higher youth homeownership rates, at the individual level, this is still expected to be largely constrained to the most economically advantaged sectors of society. In fact, notwithstanding higher overall levels, related research suggests such contexts may have more unequal homeownership access. Lersch and Dewilde (2015) find that, looking across European countries, it is in those housing contexts that are the most marketised where individual employment insecurity has the strongest negative effect on property purchase. Even in less-marketised contexts, trends towards the continued hegemony of financialisation and welfare residualisation have again pointed towards the growing salience of labour market positions and employment security (Aalbers 2008; Forrest and Hirayama 2015). While growing influence of familialism on housing trajectories might replace contexts of traditionally strong state support (i.e. Hochstenbach and Botterman 2017), this raises further issues of the reproduction of inequalities across generations and new associated insecurities for those having neither individual economic security nor recourse to parental resources.

The empirical analysis presents initial evidence across Europe of a significant correlation at the macro-level in terms of degrees of labour market inequality and insecurity in entry to homeownership for young adults. Taken in conjunction with the evidence on continuing trends towards constrained labour market opportunities and an unlikely—or undesirable—return to deregulated mortgage credit expansion, this fundamentally questions the likelihood of any resumption of homeownership growth or notions of widespread owner-occupation as a housing solution for the masses. These exploratory results encourage more detailed future empirical research into the link between underlying transformations of the labour and housing markets and how this results in diverging homeownership opportunities for current and future generations of households.

## Discussion and conclusions

While not negating variations that remain across countries, the analyses bring attention to common labour and housing market trends across the sample of European countries. The evidence presented points to a future for most European countries in which continued trajectories at levels that may be referred to as ‘mass homeownership’ are unlikely. In the years since the financial crisis of 2007, there has been a realisation that homeownership has become increasingly unattainable for large sectors of the population. Indeed, in many advanced economies—both within and beyond Europe—the secular expansion of the preceding decades alongside the ideological optimism in a socio-political project of widespread homeownership, has given way to stagnation or decreases. While older generations have sometimes been shielded by these transformations, stark reductions in homeownership entry have been evident among younger cohorts in many countries. Nonetheless, there has often been an implicit or explicit assumption that such trends are largely a product of the recent economic downturn so that an eventual post-GFC recovery will see rebounding opportunities where homeownership once again becomes widely accessible. Even if homeownership may persist as a majority tenure—considering that many who previously entered homeownership will remain owner-occupiers for years to come—where large proportions of younger generations are increasingly excluded from property ownership, we contend such a divided housing sector can no longer describe notions of mass homeownership. The argument presented here is that expectations of a return to growth have neglected fundamental longer-term labour market transformations, only partly masked by a regime of unstable credit expansion to riskier and riskier households in the pre-crisis ‘boom’ years. Across advanced economies, evidence on labour market transformations reveal long-term shifts towards growing inequality and increases in precarious employment contracts—underscored by the reduction in wage shares accruing to labour. Younger cohorts appear especially affected; their position being characterized by growing income poverty and decreasing employment participation and thus an on-going reduction in the well-paid and stable jobs required for taking on mortgage credit and entering homeownership. These fundamental changes to the labour market clearly challenge any notion of a future ‘return to normal’ characterised by widespread entry to homeownership.

Notwithstanding longer-term labour market deterioration, homeownership rates in many advanced economies actually expanded in the mid-noughties (Andrews and Caldera Sanchez 2011). This apparent misalignment can be credibly explained by the processes of financialisation and especially the expansion of credit and relaxation of mortgage requirements. The greater availability of credit meant that more and more households were able to take on larger amounts of private debt to finance home purchase—and other consumption and investment—even in the face of growing employment insecurity (Andrews and Caldera Sanchez 2011; Crouch 2009). The GFC thus provided a double-whammy in both restricting this unsustainable buffer against labour market transformations and further catalysed worsening labour conditions for those already in more precarious conditions. The immediate future—even given sustained recovery from the GFC—is thus one in which homeownership throughout many advanced economies is likely to be less widely spread through their populations. Although the housing positions of existing owners, by virtue of having paid off all or part of their housing loans, have often been sheltered from the impact of reductions in income and job security, the gradually decreasing flow of new entrants into homeownership can be expected to amplify the erosion of homeownership sectors. The notion of a Europe of homeowners may give way to housing systems characterised by greater diversity. But—and this is key—it is diversity, not as a response to consumer demands for non-owning options, but following from growing tranches of market weak consumers.

The consequences are wide reaching. Housing careers are central to life-course trajectories with, in many contexts, homeownership epitomizing a realisation of full adulthood independence. Stunted or unstable housing careers may have significant impacts on current quality of life, potential future economic wellbeing, and other spheres of adulthood transition (see Arundel and Lennartz 2017; Arundel and Ronald 2016; Buchmann 1989) with residential independence and homeownership shown to be crucially bound-up with other key stages in life-course trajectories, such as family formation and fertility (Mulder and Billari 2010; Vignoli et al. 2013). These housing dynamics take on special importance in the context of homeownership as an element in life-cycle investment strategies and notions of ‘asset-based welfare.’ In the face of state welfare residualization, accumulating private housing equity has become a major means towards financial security (Doling and Ronald 2010; Saunders 1990; Conley and Gifford 2003). Widespread homeownership was explicitly or implicitly promoted as a means of shifting responsibility onto private households and the market (Forrest and Hirayama 2009) with property purchase seen as the most suitable means of government-supported saving (Doling and Ronald 2010). The shifts towards individualized asset-based welfare have left those shut out of the housing market with increasingly diminished alternative resources. Even postponement can have significant outcomes on how long households can accumulate potential benefits from homeownership (Andrew 2012; Dewilde and Delfani 2012). Nonetheless, such trends of decreasing homeownership opportunity seem to coincide especially in those contexts where ideas of asset-based welfare have been given strongest currency (Lennartz et al. 2016).

There is a further possibility that the developing housing systems not only reflect disparity across consumers but will themselves act as a stimulus to inequality. Increasing divergence in labour market opportunities and homeownership access has seen housing wealth become a key dimension of rising inequality (Allegré and Timbeau 2015; Arundel 2017). In the UK, for example, recent empirical research has shown clear upturns in housing wealth inequality with both significant inter—as well as intra-generational divergences (Arundel 2017). The post-crisis years and changes to mortgage credit markets, have exacerbated these disparities. Forrest and Hirayama (2015, p.237) assert “the homeownership systems which have emerged from the crises are ones which favour the financially privileged—the *primes* rather than the subprimes.” Labour market polarisation has thus become amplified in the property market. One crucial dimension has been that a limited sector of the population has increasingly not only been successful in owning their own property but have further purchased other people’s homes—what may be termed a rising ‘generation landlord’ (Ronald et al. 2016).^7^ Taken together, such dynamics reflect Piketty’s (2014) central thesis of inequalities driven by rising returns from capital exceeding returns from labour.

These interrelated labour and housing market transformations have been spurred on by common shifts towards growing financialisation and neo-liberal marketization. This points to a contradiction at the heart of homeownership sectors in many developed countries. Increasingly financialised housing markets, the growth of mortgage credit and a relaxation of down-payment constraints both motivated home purchase and effectively moved the tenure down the income distribution (Andrews and Caldera Sanchez 2011). While expansion of credit may have enabled many to purchase property, interrelated processes of globalisation and financialisation have also been principal factors in growing flexibilisation, precarity and disparity in the labour market. A financialised housing market has likely spurred continued housing price increases (OECD 2011a, b) while excluding those without the stable and well-paying jobs upon which this homeownership access has been predicated. It is clear that the ‘positive’ side of financialisation in access for property loans has its limits as increasingly loose credit restrictions led to greater exposure to financial risk and the potential for individual default. On the macro-level, this can lead to economic disruptions as exemplified by the subprime crisis that triggered the GFC. The lessons of the crisis itself have not seemingly provided an alternative pathway to homeownership nor evidence of a halt of neoliberal processes (see Brenner et al. 2010; Aalbers 2013). As Forrest and Hirayama note, “the crisis of 2007–2008 did not mark a shameful retreat of neoliberal ideas but rather their reassertion in different forms adjusted for new times and new contexts” (2015, p.7). While new financial regulation may have limited mortgage access to many households, credit has seemingly refocused to those already most successful (Cunliffe 2014; Kemp 2015)—i.e. the emerging landlord class in some housing markets (Arundel 2017; Ronald et al. 2016).

Beyond growing disparities on the housing market—epitomized by notions of a ‘generation rent’ versus a ‘generational landlord’—labour market and housing system transformations may very well undermine the viability of the economic system upon which it is predicated. What is happening to homeownership can be seen as part of a wider crisis of capitalism. While lower wages may help individual firms to compete by driving down production costs and provoke similar measures among competitors, wages are not only a component of production, but also the source of demand. More generally, wider inequality will have a negative impact on aggregate demand and economic growth (see, for example, Mason 2015; Stiglitz 2012; Palley 2012). Homeownership dynamics may serve as a canary in a coal mine, where the multiple prerequisites to home-purchase become more difficult to align as changing labour markets and financialisation processes erode income and job stability across a large middle-class sector of the population and most clearly among the younger generations who comprise the future of housing market demand.

## Footnotes

1 The number of countries varies slightly across analyses depending on the availability of harmonized data.

2 This had an especially large impact in Eastern European countries where the post-communism transition saw large-scale privatisation of former state housing resulting in the current very high rates of homeownership.

3 The FSB was established in 2009 with membership of the G20 countries, the European Commission, IMF, OECD and the Bank for International Settlements. Its role is to monitor and make recommendations about global financial systems especially in prudential regulations governing capital and liquidity requirements.

4 Varying lengths of years of harmonised data is available for different measures. For some measure, where longer-term harmonized data is only available for EU15 countries, both country samples are shown. Considering that the full available sample trends matches the core EU15 data, the long-term pattern is likely indicative for the full EU average.

5 In the indicators analysed there are some variations in age categories of younger adults reflecting differences across available data sources. Nonetheless, the indicators capture key populations across the period of young adulthood and the differences are secondary to the focus on changes over time.

6 Based on OECD definitions, not in permanent employment includes those unemployed, in ‘casual’ employment, or with short-term contracts of less than a year in duration.

7 The accumulation of secondary properties for rental has been dramatic over recent years in some countries. In the UK, landlords grew from just over 500,000 in 1991 to 2.12 million in 2012 (Ronald et al. 2016) with similar growth in private rental and landlordism in the USA and in Australia (National Low Income Coalition 2011; Forrest and Hirayama 2015).
